# Towards next generation white LEDs: optics-electronics synergistic effect in a single-layer heterophase halide perovskite

**DOI:** 10.1038/s41377-021-00488-8

**Published:** 2021-03-01

**Authors:** Andrey L. Rogach

**Affiliations:** Department of Materials Science and Engineering, and Centre for Functional Photonics (CFP), City University of Hong Kong, Hong Kong SAR, PR China

**Keywords:** Optics and photonics, Physics

## Abstract

A novel concept of the *heterophase optics-electronics synergistic effect* has been demonstrated in a single-layer α/δ-heterophase perovskite CsPbI_3_ in order to realize white LEDs featuring only one broadband emissive layer.

Light sources have been under steady development all the way throughout the human existence, from ancient times to the modern technology, and sources of lighting experienced changes from fire to electricity. Since the dawn of the first semiconductor light-emitting diodes (LEDs), these devices showed many advantages over traditional incandescent or fluorescent lighting, such as high luminous efficiency, energy saving, and color quality^1^. Traditional approach to realize white LEDs (WLEDs) relies on the down-conversion of light emitted by a combination of red (R) and green (G) phosphors excited by blue (B) light, or on electrical excitation of RGB emitters arranged into LED arrays^2,3^. However, the use of the rare-earth elements in down-conversion devices faces issues of high cost and scarcity, and the most common blue-emitting component of such LEDs (GaN) is fabricated through rather expensive epitaxial growth techniques. Thus, exploring new technologies for lighting which may lead to more cost-efficient WLEDs is highly desired.

Halide perovskites have recently attracted a lot of attention as promising monochromatic bright emitters able to offer high-quality light in LEDs^4^. External quantum efficiencies (EQE) of perovskite-based green and red LEDs already exceeded 20%^5,6^. Despite a great progress on monochromatic perovskite LEDs, development of perovskite WLEDs has been rather slow, so far, and mostly relied on the doping with other elements^7,8^. High rate of anion diffusion in mixed-halide perovskite materials, combined with non-balanced degradation under working conditions severely hinder the use of the LED arrays due to the rapid change of emission color^9^. Moreover, efficiencies and stabilities of blue perovskite LEDs still lag behind their red and green counterparts^10^, so that more efforts are needed for their development. Very recently, Chen and co-workers used an advanced device structure that could efficiently suppress the trapped optical modes^11^. It comprised a layer of red-emitting perovskite nanocrystals acting as a down-converter, coated on a blue perovskite LED with an ultrathin transparent top electrode. An efficient extraction of the trapped waveguide mode and surface plasmon polariton mode in the blue LED was realized, leading to over 50% improvement in a light-extraction efficiency, and also efficient blue-to-red light conversion, resulting in a complementary white light emission with a high EQE of 12%.

In another approach, Zeng’s group at Nanjing University of Science and Technology (NJUST) in collaboration with D. Ginger at the University of Washington proposed a new concept of WLEDs relying on a *single emissive layer* of CsPbI_3_ perovskite which combined α and δ-phases (Fig. 1a)^12^. EQE and current efficiency of these WLEDs which were conveniently fabricated by all-solution processing and featured only one broadband perovskite emitting layer reached 6.5% and 12,200 cd/m^2^, respectively. Importantly, by adjusting the annealing processes of α-CsPbI_3_, the ratio of the two phases could be controlled to tailor the color temperature of white emission (Fig. 1b).

**Fig. 1:**
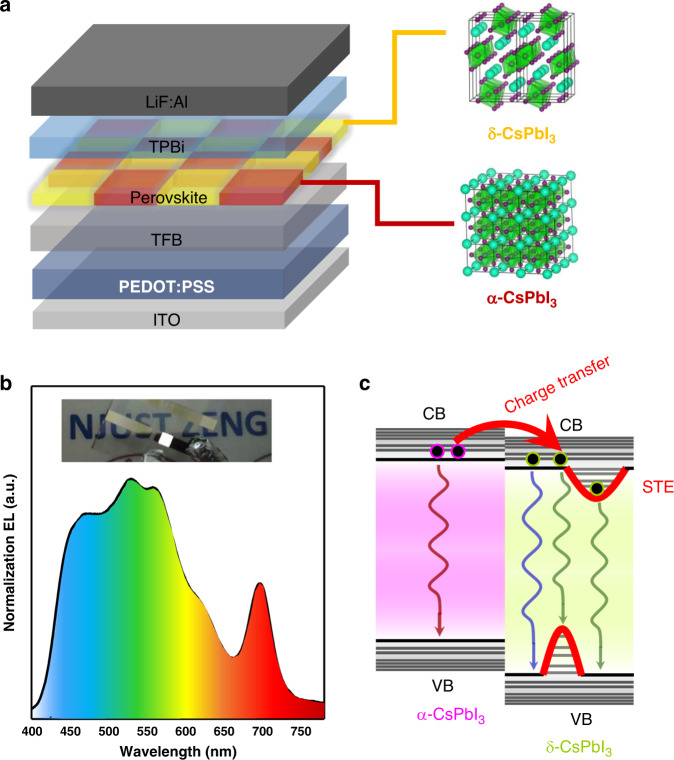
WLEDs relying on a single emissive layer of a heterophase cesium lead halide perovskite. **a** Scheme of the WLED structure relying on a synergistic effect of α- and δ-CsPbI_3_ perovskite heterophases. **b** Broad electroluminescence spectrum of the WLED; photograph in the inset shows its uniform white emission. **c** Charge transfer and recombination mechanism operative in the α/δ-CsPbI_3_ heterophase WLED (STE stays for “self-trapped exciton”)

The major idea of operation of these WLEDs relied on a synergistic effect between α/δ-CsPbI_3_ phases (Fig. 1c): charge carrier injection occurred in the α-CsPbI_3_ phase, which was followed by charge transfer from α- to δ-phases, and white light emission by δ-CsPbI_3_. The interfacial states which are energetically closer to the valence band (VB) level of α-CsPbI_3_ assisted hole transfer from α-CsPbI_3_ to δ-CsPbI_3_, while the conduction band (CB) alignment between α-CsPbI_3_ and δ-CsPbI_3_ phases allowed for an optimum electron injection and an efficient radiative recombination. The authors have termed this mechanism “*heterophase optics-electronics synergistic effect*”.

The strategy used in ref. ^12^ relying on a single emitting perovskite layer to achieve white emission can greatly decrease the cost of production of WLEDs, and further push the development of next-generation flexible displays and lighting. This approach, as well as the one suggested in ref. ^11^ may also have important implications not only for LEDs but for perovskite optoelectronics in general, such as for solar cells and displays.
